# Neuropsychiatric symptoms and their impact on cognitive functioning in patients with parkinson's disease: A systematic review of the literature

**DOI:** 10.3758/s13415-026-01424-2

**Published:** 2026-03-26

**Authors:** Barbara Blasutto, Giulia Matrone, Byron Creese, Francesco Fattapposta, Maria Casagrande

**Affiliations:** 1 Department of Psychology– “Sapienza”, University of Rome, Via dei Marsi 78, 00185 Rome, Italy; 2 Department of Dynamic and Clinical Psychology, and Health Studies – “Sapienza”, University of Rome, Via degli Apuli 1, 00185 Rome, Italy; 3 Department of Psychology, College Health Medicine and Life Sciences, Brunel University of London, London, UK; 4 Department of Human Neuroscience, “Sapienza” University of Rome, Viale dell’Università 30, 00185 Rome, Italy

**Keywords:** Parkinson's disease, Neuropsychiatric symptoms, Mild behavioral impairment, Cognitive decline, Systematic review

## Abstract

Parkinson's disease (PD) is a progressive, neurodegenerative disorder that affects the dopaminergic system and is characterized by motor and nonmotor symptoms, which affect the quality of life. Among these, neuropsychiatric symptoms (NPS), such as depression, hallucinations, and apathy, are common and can accelerate cognitive decline. Although the association between some specific NPS (e.g., apathy) and cognitive functions has been investigated, no review has systematically examined the relationship between the whole of NPS and cognitive functions in PD patients without dementia. Therefore, the purpose of this study was to analyze the relationship between NPS taken together and cognitive impairment in patients with PD. According to the PRISMA-Statement, this systematic review critically examined the difference in cognitive performance between patients diagnosed with idiopathic PD with and without NPS. Results were classified according to the cognitive domain evaluated. Eleven studies met the eligibility criteria. The results showed that PD patients with NPS showed impaired performance on cognitive tasks compared with those without NPS, as well as in longitudinal studies. These results were also confirmed when the different domains were considered separately. Despite the limited number of included studies, the importance of assessing NPS in their totality rather than individual behavioral symptoms emerges. Clinical manifestations may vary among individuals and across different stages of the disease. Therefore, assessing the presence of these symptoms and the timing of their onset would be appropriate and may give the clinician important insights into the possible course and management of the disease.

## Introduction

Parkinson’s disease (PD) is a chronic, progressive, neurodegenerative disorder with a wide range of motor and nonmotor symptoms. Neuropsychiatric symptoms (NPS) play a central role in determining overall disability, clinical progression, and quality of life (Gökçal et al., 2017). Disorders, such as depression, anxiety, apathy, irritability, hallucinations, and sleep disturbances, have been observed at all stages of PD and often are underestimated or inadequately treated (Mueller et al., 2018; Schneider et al., 2008).

The high prevalence of NPS and their interaction with cognitive aspects of PD have drawn increasing attention in the scientific literature. It has emerged that these symptoms can negatively affect cognitive functioning, particularly in domains, such as attention, executive functions, and working memory (Aarsland et al., 2009a, b). While cognitive impairment in PD has traditionally been linked to advanced stages of the disease, the presence of NPS may indicate an early risk factor or modulator of cognitive decline trajectory (Herman et al., 2015).

From a neurobiological perspective, there is a growing consensus that NPS and cognitive alterations share common pathophysiological mechanisms, including involvement of frontostriatal circuits, dopaminergic pathways, and serotonergic and noradrenergic projections (Aarsland et al., 2009a, b; Schneider et al., 2008). These structural and functional overlaps may explain why affective symptoms frequently coexist with impaired cognitive abilities in patients with Parkinson’s disease.

For the clinical assessment of neuropsychiatric symptoms, the reference literature commonly employs standardized tools, among which the Neuropsychiatric Inventory (NPI) is one of the most widely used instruments for detecting behavioral and affective symptoms, such as anxiety, depression, apathy, and psychotic symptoms. The assessment of depression and anxiety is often specifically conducted by using the Geriatric Depression Scale (GDS-15) and the State-Trait Anxiety Inventory (STAI), respectively, sensitive to subclinical depressive symptoms and state and trait anxiety components. Additionally, Part I of the Movement Disorder Society–Unified Parkinson's Disease Rating Scale (MDS-UPDRS-I) is a complementary measure of nonmotor symptoms, including neuropsychiatric symptoms. It is essential for a comprehensive clinical overview of patients (Mueller et al., 2018; Aarsland et al., 2009a, b).

Cognitive functioning in the PD population is generally assessed by using global screening tools, such as the Mini-Mental State Examination (MMSE), which has limited sensitivity to early executive deficits (Zadikoff et al., 2008; Hoops et al., 2009), and the Montreal Cognitive Assessment (MoCA), preferred in clinical and research settings for its ability to detect early changes in cognition (Dalrymple-Alford et al., 2010; Skorvanek et al., 2018). The International Parkinson and Movement Disorder Society (MDS) also recommends the SCales for Outcomes in PArkinson’s disease-COGnition (SCOPA-COG) (Marinus et al., 2003), and its task force advises using at least two tests per cognitive domain for Level II PD-MCI diagnosis (Litvan et al., 2012).

Using differentiated tools with specific psychometric properties reflects the clinical and cognitive complexity of PD and highlights the need for a multidimensional approach to capture subtle manifestations that are often predictive of subsequent cognitive decline. Considering these factors, this systematic review focuses specifically on the combined impact of all NPS as a whole on cognitive functioning in patients with idiopathic PD, excluding those with overt dementia. Specifically, the review will determine whether the overall presence of NPS negatively influences cognitive functioning in patients with idiopathic PD compared with patients without such symptoms. To this end, the present study focused exclusively on research evaluating NPS as a composite construct, rather than examining its individual domains separately. Although specific symptoms, such as apathy, hallucinations, and impulse control disorders, have frequently been linked to poorer cognitive functioning (Aarsland & Kramberger, 2015; Santangelo et al., 2017; Samundra et al., 2016), the overall impact of the combined presence of NPS on cognition has not been thoroughly investigated. We believe that considering NPS collectively better captures the overall neuropsychiatric burden that may reflect widespread neuropathological changes or common underlying mechanisms that single symptoms cannot fully represent. This global burden may be a stronger predictor of functional decline, caregiver distress, and risk of progression, as has been observed in other conditions, such as mild cognitive impairment (Martin & Velayudhan, 2020).

## Methods

### Search strategies

In accordance with the Preferred Reporting Items for Systematic Reviews and Meta-Analyses (PRISMA) 2020 Statement (Page et al., 2021), an international literature review and meta-analysis were conducted and registered on Prospero (ID: CRD42025640916). Articles published in peer-reviewed journals were selected by using PsycINFO, MEDLINE, Scopus, and Web of Science. The final search was conducted on October 13, 2025. Results were restricted to publications in English, Italian, Spanish, and German. The included studies were conducted on populations of humans, without restrictions of gender and ethnicity, which considered subjects with idiopathic PD with and without NPS who underwent at least one cognitive assessment.

The script used included the following keywords:

- Parkinson* AND (“neuropsychiatr* symptom*” OR NPS OR NPI OR NPI-Q OR BPSD OR “Behavio* psycholog* symptom*” OR “mild behavioral impairment" OR "mild behavioural impairment")
- AND (neuropsycholog* OR cognition OR “cognitive function*” OR “cognitive skill*” OR “cognitive abilit*” OR “cognitive impairment*” OR “cognitive dysfunction*” OR “cognitive problem*” OR “cognitive profile*” OR “cognitive deficit*” OR memory OR Attention* OR Executive OR speed OR language OR visuospatial OR visuoconstruction* OR fluency OR learning).

### Eligibility criteria

The articles identified through the literature search were evaluated and selected based on the following criteria: subjects with PD with NPS and without dementia (Parkinson’s dementia (PDD), as defined by Emre et al., 2007), the research had to investigate the impact of these symptoms on at least one cognitive function or overall cognitive functioning and compare the results either with a population of healthy subjects of comparable age, or with PD but without NPS and PDD, or with the same group but at a later date. Studies with control groups consisting of patients with other neurodegenerative diseases were excluded; however, the presence of mild cognitive impairment (MCI) or subjective cognitive decline was not considered an exclusion criterion, because these conditions are not associated with significant interference in activities of daily living (Litvan et al., 2012). Regarding NPS, only studies that assessed these symptoms collectively were included, excluding those that considered them individually, as defined by established NPS frameworks (e.g., Neuropsychiatric Inventory or Mild Behavioral Impairment (MBI) domains). Studies that considered other neurodegenerative diseases, parkinsonism, psychiatric disorders, PDD, or Alzheimer’s dementias or other type of dementia, as well as studies without cognitive assessment, were excluded. Additionally, studies that only included neuroimaging, genetic, or other types of publications were excluded.

**Fig. 1:**
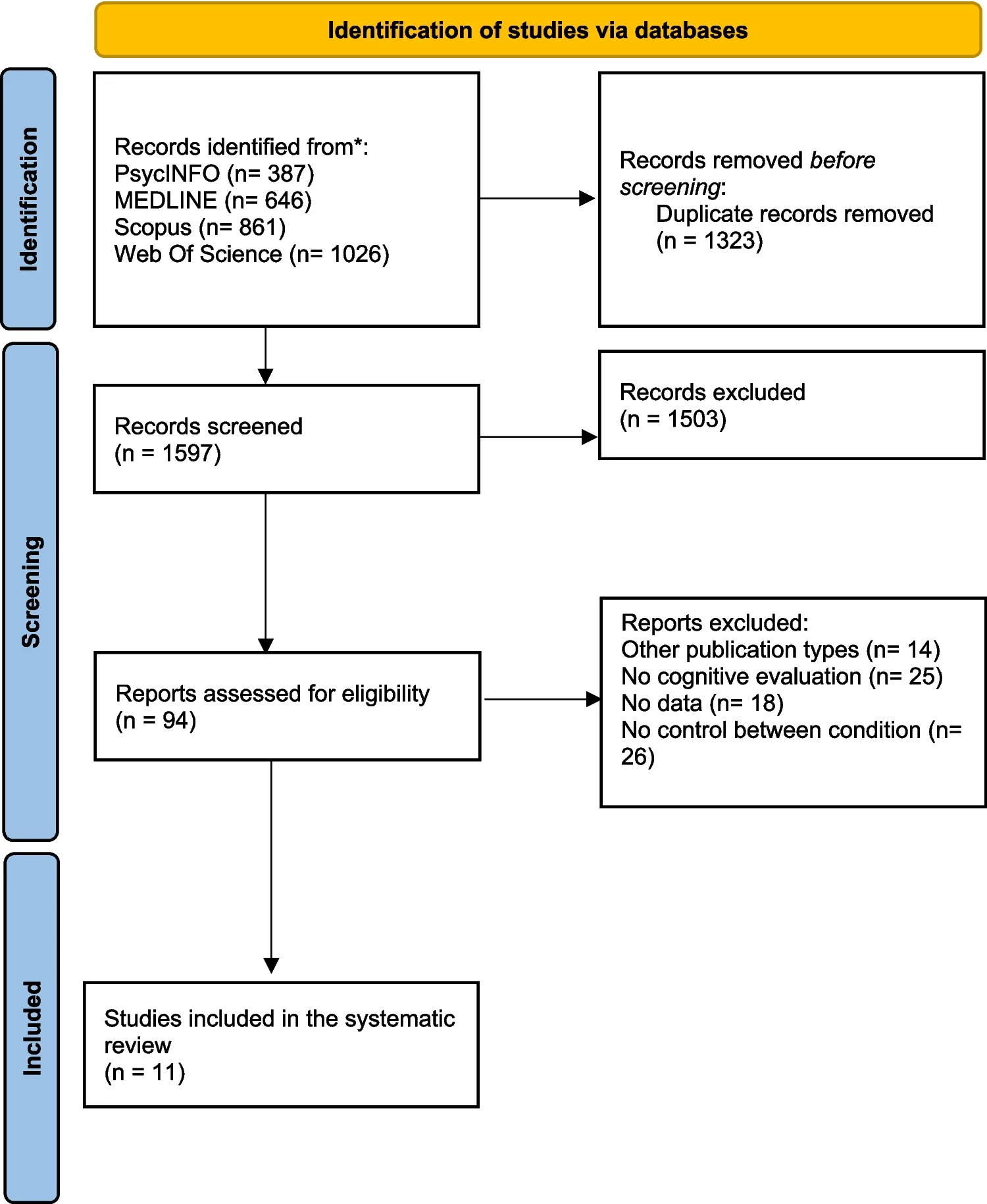
PRISMA 2020 flow diagram-included studies

The studies were downloaded onto the Mendeley platform, which, after removing duplicates, permitted an initial reading and the selection of articles based on title and abstract. Subsequently, a more comprehensive analysis was conducted by reading the full texts, which enabled the exclusion of further studies (Fig. 1).

### Data collection process

For each selected article, information relevant to the study was extracted and analyzed to ensure a structured and consistent comparison between the different studies. Specifically, the following information was recorded for each study: authors and year of publication; total number of subjects; and division into groups, distinguishing between patients with Parkinson's disease with or without NPS, as well as the healthy control group, if present.

Demographic data were also collected, including the average age of participants, the relative standard deviation, the average number of years of schooling, and the percentage of female subjects. The type of study was classified as cross-sectional, longitudinal, or retrospective, depending on the experimental design adopted by the authors. For the clinical characterization of the PD subjects, the Hoehn and Yahr (H&Y) scale and the UPDRS III score were reported and, where available, the disease duration. We also reported the daily equivalent dose of levodopa—a parameter useful for estimating the current dopaminergic therapy. Neuropsychiatric symptoms assessment was conducted by analyzing the scales used in the studies to measure these symptoms. At the same time, we considered the cognitive tests, paying particular attention to the domains explored.

Finally, the main results from each study were summarized, particularly referencing any associations between NPS and cognitive functioning. All information was organized and systematized in Table 1.

**Table 1:** Characteristics of the studies

Authors; yr	No. subjects	Age (SD)	Education (yr SD)	% female	Study design	Valuation of NPS	LEDD (mg/die)	H&Y	UPDRS III	Disease duration	Cognitive domain	Main results
Lee et al., 2023	PD-converter: 41 PD-non converter: 297	PD-converter: 71.5 (8.2) PD-nonconverter: 69.7 (7.7)	PD-converter: 8.9 (4.8) PD-nonconverter: 10.0 (4.8)	PD-converter: 41.5%, PD-nonconverter: 48.5%	Retrospective study	NPI	PD-converter: 387.5 (393.0) PD-nonconverter: 249.9 (308.0)	n/a	PD-converter: 28.0 (8.7) PD-nonconverter: 21.8 (8.7)	PD-converter 2.4 (±1.5) PD-nonconverter 2.2 (±1.7)	Global cognitive functioning (K-MMSE), Verbal Memory (SVLT), Executive function (Stroop Color Reading Test; DSB), Language (K-BNT; Phonemic COWAT), Visuospatial ability (RCFT copy).	The "mood" cluster was associated with lower scores on the SVLT and Stroop Color Reading Test; the "hyperactivity" cluster was associated with lower scores on the K-BNT, RCFT copy, and SVLT; and the "psychotic" cluster was associated with lower scores on the RCFT copy.
Weintraub et al., 2015^**a**^	PD (untreated): 423 HC: 196	PD: 61.7 (10.6) HC: 60.8	PD: 13.8 (2.8) HC: 14.2 (2.6)	PD: 35% HC: 36%	Transversal study	GDS-15, STAI, QUIP, MDS-UPDRS I	-	n/a	PD: 20.9 HC: 1.2	n/a	Global cognitive functioning (MoCA), Memory (HVLT-R); Visuospatial ability (Benton Judgment of Line Orientation); Attention and processing speed (Symbol-Digit Modalities Test); Executive function (Letter-Number Sequencing- ing17 and semantic (animal) fluency).	No significant association emerged between cognitive tasks and the severity of anxiety, depression, or apathy.
Meng et al., 2023^**a**^	PD: 423	n/a	n/a	n/a	Longitudinal study	GDS-15, STAI, QUIP, MDS-UPDRS Part I	n/a	n/a	n/a	n/a	Global cognitive functioning (MoCA), Memory (HVLT-R); Visuospatial ability (JOLO); Attention and processing speed (SDMT); Executive function (Letter Number Sequencing, Semantic Fluency Test).	PD patients with NPS in follow-ups show lower cognitive functioning than PD patients without NPS. In particular, depressive symptoms were associated with lower scores on the MoCA, HVLT-R, SDMT, Semantic Fluency test, and JOLO; apathy was associated with lower scores on the MoCA and SDMT; psychosis was associated with lower scores on the MoCA; anxiety was associated with lower scores on the HVLT-R, SDMT, and Semantic Fluency.
Lang et al., 2020*	PD-MBI: 21 PD-noMBI: 53 HC: 28	PD-MBI: 71.8 (6.4), PD-noMBI: 70.4 (5.8), HC: 69.8 (6.7)	PD-MBI: 13.9 (3.3), PD-noMBI: 15.2 (2.5), HC: 16.2 (2.8)	PD-MBI: 28.6% PD-noMBI: 35.8% HC: 53.6%	Transversal study	MBI-C	PD-MBI: 834.0 (412.0) PD-noMBI: 742.1 (369.9)	I-III	PD-MBI: 22.9 (8.0) PD-noMBI: 17.2 (11.0)	PD-MBI: PD-noMB:	Global cognitive functioning (MoCA); Memory (HVLT, WMS-IV Logical Memory, RCFT recall trials); Visuospatial ability (HVOT/Benton JOLO, RCFT Copy trial); Attention (Trail Making Test A, WMS-IV Symbol Span, WAIS-IV Digit Span FWD); Executive function (STROOP Colour-Word, Brixton Spatial Anticipation, Hayling Sentence Completion, Trail Making Test B, Clock Drawing Test - Command); Language (Boston Naming Test, Semantic Fluency - Animali/Azioni).	PD-MBI patients had worse cognitive abilities in all domains compared to HC, and had lower scores in MoCA, attention, visual abilities, and memory compared to PD-noMBI.
Yoon et al., 2019	PD-MBI: 20 PD-noMBI: 40 HC: 29	PD-MBI: 71.3 (6.5) PD-noMBI: 70.2 (6.2) HC: 68.7 (5.9)	PD-MBI: 14.4 (3.2) PD-noMBI: 15.4 (2.6), HC: 16.7 (2.6)	PD-MBI: 25% PD-noMBI: 33% HC: 52%	Transversal study	MBI-C	PD-MBI: 910.7 (430.6) PD-noMBI: 730.9 (342.5)	II-III	PD-MBI: 22.0 (8.4) PD-noMBI: 16.7 (9.1)	PD-MBI: PD-noMBI:	Global cognitive functioning (MoCA); Executive function (Trail Making Test B, Stroop Color-Word, Hayling Sentence Completion, Brixton Spatial Anticipation, Digit Span Backward e Sequencing di WAIS-IV, Clock Drawing Test - Command, Letter Fluency - FAS); Attention (Trail Making Test A, WAIS-IV Digit Span Forward, WMS-IV Symbol Span); Language (Boston Naming Test, Semantic Fluency - Animali/Azioni); Memory (Hopkins Verbal Learning Test, Rey-Osterrieth Complex Figure Test - Delayed Recall, WMS-IV Logical Memory); Visuospatial ability (Hooper Visual Organization Test, Rey-Osterrieth Complex Figure Test - Copy)	The PD-MBI group obtained significantly lower scores than PD-noMBI and HC in the MoCA. PD-MBI showed deficits in all cognitive domains compared to PD-noMBI and HC, while no significant differences emerged between PD-noMBI and HC. Furthermore, the severity of neuropsychiatric symptoms (total MBI-C) was negatively correlated with performance in the domains of attention, executive functions, memory, and visuospatial skills.
Oh et al., 2021	PD-NPS: 98 PD-no NPS: 58	PD-NPS: 72.0 (8.5) PD-no NPS: 71.2 (8.3)	n/a	PD-NPS: 41.8% PD-no NPS: 51.7%	Transversal study	NPI	n/a	PD-NPS: 1.9 (0.7) PD-no NPS: 1.5 (0.6)	PD-NPS: 18.3 (8.9) PD-no NPS: 14.8 (9.3)	PD-NPS: 1.9 (±0.7) PD-no NPS: 1.5 (±0.6)	Global cognitive functioning (MMSE, CDR, GDS)	The PD-NPS group obtained significantly lower scores on the MMSE than the PD-noNPS group. No significant differences emerged in the CDR and GDS between the two groups.
Yoon et al., 2021	PD-MBI: 21 PD-noMBI: 38 HC: 26	PD-MBI: 70.9 (6.6) PD-noMBI: 69.9 (6.3) HC: 68.6 (6.1)	PD-MBI: 14.1 (3.4) PD-noMBI: 15.4 (2.6) HC: 16.4 (2.8)	PD-MBI: 24% PD-noMBI: 39% HC: 62%	Transversal study	MBI-C	PD-MBI: 882.8 (478.0) PD-noMBI: 681.1 (272.2)	II-III	PD-MBI: 21.9 (8.4) PD-noMBI: 15.3 (7.8)	PD-MBI: PD-noMBI:	Global cognitive functioning (MoCA); Executive function (Wisconsin Card Sorting Test - WCST, Trail Making Test B, Stroop Color-Word Test); Memory (Hopkins Verbal Learning Test - HVLT, Rey-Osterrieth Complex Figure Test - Delayed Recall, Logical Memory - WMS-IV); Attention (Trail Making Test A, WAIS-IV Digit Span Forward); Visuospatial ability (Rey-Osterrieth Complex Figure Test - Copy).	The PD-MBI group performed significantly worse than PD-noMBI and HC in the MoCA, with no significant differences between PD-noMBI and HC. PD-MBI performed significantly worse in the WCST than HC; there were no significant differences between PD-noMBI and HC. In addition, PD-MBI made significantly more non-perseverative errors than PD-noMBI.
Kulisevsky et al., 2008	PD-ND: 1351	70.6 (9.1)	5.65 (4.94)	44.4%	Transversal study	NPI HADS	n/a	I: 15.1% II: 42.2% III: 31% IV: 11% V: 0.7%	n/a	n/a	Executive function (Semantic Fluency, Phonemic Fluency, Alternating Fluency)	PD-NPS patients performed significantly worse on verbal fluency tests than PD-noNPS patients.
Dlay et al., 2020	PD: 212 HC: 99	PD: 66.2 (10.1) HC: 67.5 (7.5)	PD: 12.5 (3.5) HC: 13.0 (3.3)	PD: 35.8% HC: 44.4%	Longitudinal study (36 month)	NPI	Baseline: 190.4 (159.9) 18 months: 413.8 (214.3) 36 months: 518.2 (273.5)	Baseline: 1.9 (0.7) 18 months: 2.2 (0.5) 36 months: 2.1 (0.6)	Baseline: PD 27.7 (12.3) 18 months: 33.3 (12.1) 36 months: 35.2 (15.0)	PD: 0.5 (±0.45)	Global cognitive functioning (MoCA)	PD-NPS patients did not show significantly different cognitive decline compared to PD-noNPS patients over the 36-month follow-up period.
Ojagbemi, 2013	PD-MCI: 12 PD-CN: 38	PD-MCI: 64.3 (9.7) PD-CN: 64.3 (9.7)	n/a	PD-MCI: 50% PD-CN: 42.1%	Transversal study	NPI	n/a	n/a	PD-MCI: 42.0 (5.8) PD-CN: 42.0	PD-MCI: >2 anni PD-CN: ≤2 anni	Global cognitive functioning (MMSE)	PD-MCI patients had a higher frequency of hallucinations and agitation than PD-CN patients. Neuropsychiatric symptoms were more severe in patients with MCI, although there was no significant difference. Furthermore, no significant correlation was found between MMSE scores and total NPI scores.
Aarsland et al., 2009a, b	PD: 175 HC: 166	PD: 67.8 (9.0) HC: 67.3 (9.1)	PD: 10.9 (2.3) HC: 11.6 (3.5)	PD: 42% HC: 40%	Transversal study	NPI	n/a	n/a	22.8 (11.2)	n/a	Global cognitive functioning (MMSE)	PD-NPS patients obtained significantly lower scores on the MMSE than PD-noNPS patients. The group with higher apathy scores showed more marked cognitive impairment than the group with predominantly mood symptoms and the group with no/mild NPS.

PD = Parkinson’s disease; PD-Converter = Parkinson’s disease patients who converted to dementia; PD-nonconverter = Parkinson’s disease patients who did not convert to dementia; PD-MBI = Parkinson’s disease with mild behavioral impairment; PD-noMBI = Parkinson’s disease without mild behavioral impairment; PD-ND = Parkinson’s disease–no dementia; PD-NPS = Parkinson’s disease with neuropsychiatric symptoms; PD-noNPS = Parkinson’s disease without neuropsychiatric symptoms; HC = healthy controls; NPS = neuropsychiatric symptoms; NPI = neuropsychiatric inventory; NPI-Q = Neuropsychiatric Inventory–Questionnaire; NPI-D = Neuropsychiatric Inventory–Distress; GDS = Global Deterioration Scale; COWAT = Controlled Oral Word Association; Digit Span Backward (DSB); GDS-15 = Geriatric Depression Scale (15 Items); CDR = clinical dementia rating; STAI = State–Trait Anxiety Inventory; HADS = Hospital Anxiety and Depression Scale; LEDD = Levodopa Equivalent Daily Dose; H&Y = Hoehn and Yahr Scale; UPDRS = Unified Parkinson’s Disease Rating Scale; UPDRS III = Unified Parkinson’s Disease Rating Scale, Part III; MDS-UPDRS = Movement Disorder Society–Unified Parkinson’s Disease Rating Scale; MoCA = Montreal Cognitive Assessment; MMSE = Mini–Mental State Examination; HVLT-R = Hopkins Verbal Learning Test–Revised; JOLO = Judgment of Line Orientation; SDMT = Symbol Digit Modalities Test; SVLT = Seoul Verbal Learning Test; K-BNT = Korean–Boston Naming Test; RCFT = Rey–Osterrieth Complex Figure Test; HVOT = Hooper Visual Organization Test; WMS-IV = Wechsler Memory Scale, Fourth Edition; WAIS-IV = Wechsler Adult Intelligence Scale, Fourth Edition; WCST = Wisconsin Card Sorting Test; QUIP = Questionnaire for Impulsive–Compulsive Disorders in Parkinson’s Disease; MBI-C = Mild Behavioral Impairment Checklist; PPMI = Parkinson’s Progression Markers Initiative; SD = standard deviation; n/a = not available

^a^This study included subjects enrolled in PPMI

^*^This study shares the group of subjects with the study by Yoon et al. (2019), already included in the analysis

### Risk of bias

The Quality In Prognosis Studies (QUIPS) was used to conduct a quality assessment of the selected studies (Hayden et al., 2013). For this systematic review and meta-analysis, we considered the following risks of bias:

1. *Study participation* to assess whether the study sample adequately represents the population of interest;
2. *Study attrition* to assess whether the available data adequately represent the study sample;
3. *Prognostic factor* (*PF*) *measurement* to assess whether PF is measured similarly for all participants;
4. *Outcome measurement* to assess whether the outcome of interest is measured similarly for all participants;
5. *Confounding factors of the study* to assess whether important measures of potential confounding were provided; and
6. *Statistical analysis and reporting* to assess whether statistical analyses are appropriate and whether all primary outcomes are reported.

**Fig. 2:**
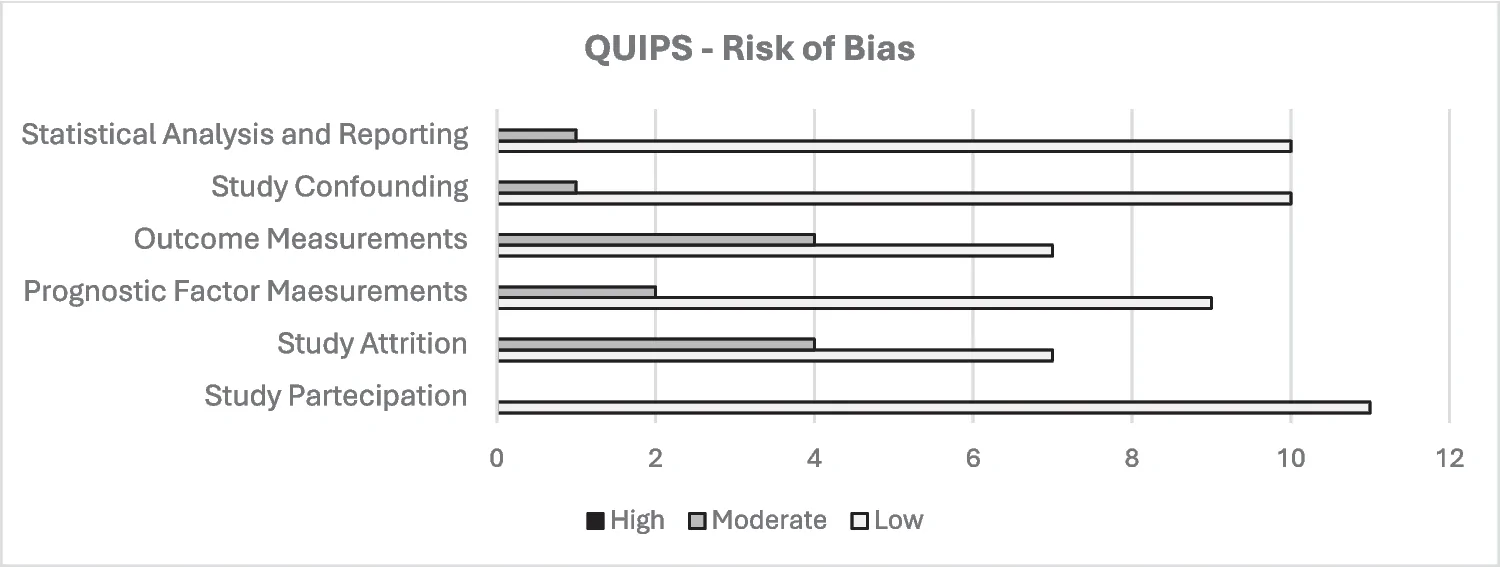
QUIPS—Risk of bias of selected studies

Figure 2 presents a general overview of the possible risks of bias in all the studies examined.

## Results

### Selection of studies

The flow chart shows the process of selecting articles from various databases and the number of studies examined, evaluated for eligibility and included in the review, with reasons for any exclusions (Fig. 1). The bibliographic search yielded 2,920 studies. After eliminating duplicates using the Mendeley program, the total was reduced to 1,597 studies. The titles and abstracts of these studies were read. After this process, 94 articles remained, and their full texts were read. After further selection, only 11 articles were included in the systematic review.

#### Bias risk of selected studies

Of the 11 studies included in the systematic review, five reported no risk of bias in the analyzed domains (Aarsland et al., 2009a, b; Yoon et al., 2019; Oh et al., 2021; Meng et al., 2023; Lee et al., 2023), five studies reported a moderate risk of bias in one of the subscales (Weintraub et al., 2015; Lang et al., 2020; Dlay et al., 2020; Yoon et al., 2021; Lee et al., 2023), and one study reports a moderate risk of bias in more than two subscales (Ojagbemi, 2013). Regarding the "study participation" factor, all studies were classified as having a low risk of bias. Seven studies present a low risk for the “study attrition” domain, while four report a moderate risk. Regarding “prognostic factor measurement,” nine studies are at low risk, and two are at moderate risk. Seven studies show a low risk, and four show a moderate risk for the “outcome measurement” factor. The "confounding" factor was considered low risk in ten studies and moderate risk in one. Finally, ten studies were assessed as low risk and one as moderate risk for the “statistical analysis and reporting” factor.

None of the studies reported a high risk of bias in any subsection. Figure 2 (QUIPS) illustrates the risk of bias for each analyzed domain.

### Demographic data

The 11 studies that met the inclusion criteria were conducted between 2008 and 2023 and involved 3,865 participants, of whom 3,321 were diagnosed with PD and 544 were HC. The average age of the PD patients ranged from 61.7 years (Weintraub et al., 2015) to 72.0 years (Oh et al., 2021), whereas the average age of the HC ranged from 60.8 years (Weintraub et al., 2015) to 69.8 years (Lang et al., 2020).

The percentage of women in the PD groups ranged from 24% (Yoon et al., 2021) to 50% (Ojagbemi, 2013). In the HC groups, it ranged from 36% (Weintraub et al., 2015) to 62% (Yoon et al., 2021). One study (Meng et al., 2023) did not report information on the participants’ gender.

Most studies adopted a cross-sectional design, while two studies (Meng et al., 2023; Dlay et al., 2020) also included a longitudinal assessment.

The studies were conducted in various geographical areas: the United States (Weintraub et al., 2015; Meng et al., 2023); Canada (Lang et al., 2020; Yoon et al., 2019, 2021); South Korea (Oh et al., 2021; Lee et al., 2023); Spain (Kulisevsky et al., 2008); the United Kingdom (Dlay et al., 2020); Norway (Aarsland et al., 2009a, b); and Nigeria (Ojagbemi, 2013).

### Measuring MBI

The assessment of NPS in PD patients was performed by using various tools across the studies included, reflecting the complexity and multidimensionality of these symptoms. The most widely used scales were the Neuropsychiatric Inventory (NPI) (Cummings et al., 1994) in its various forms, the Mild Behavioral Impairment Checklist (MBI-C) (Ismail et al., 2017), and the MDS-UPDRS Part I (Goetz et al., 2008). Other studies have used various domain-specific instruments, including the Non-Motor Symptoms Scale (Chaudhuri et al., 2007); the Hamilton Anxiety Rating Scale (HAM-A) (Hamilton, 1959); the Hamilton Depression Rating Scale (HAM-D) (Hamilton, 1960); the Apathy Scale (AS) (Starkstein et al., 1992), the Geriatric Depression Scale—15 Items (GDS-15) (Yesavage & Sheikh, 1986), the Hospital Anxiety and Depression Scale (HADS) (Zigmond & Snaith, 1983), and the State-Trait Anxiety Inventory (STAI) (Spielberger et al., 1971).

The NPI-Q (Kaufer et al., 2000) has been used in numerous studies (Lee et al., 2023; Oh et al., 2021; Kulisevsky et al., 2008; Aarsland et al., 2009a, b; Ojagbemi, 2013; Dlay et al., 2020) to assess the presence and severity of 12 neuropsychiatric domains: delusions; hallucinations; agitation/aggression; depression/dysphoria; anxiety; euphoria; apathy; disinhibition; irritability/lability; aberrant motor behavior; sleep and eating disorders. The NPI-D version, adopted for example by Dlay et al. (2020), assesses the level of distress perceived by the caregiver. The MBI-C (Ismail et al., 2017), used by Lang et al. (2020) and Yoon et al. (2019, 2021), is the reference tool for identifying mild behavioral impairment, a late-onset syndrome characterized by behavioral and personality changes associated with cognitive decline. This 34-item questionnaire assesses behavioral changes according to five domains: apathy/motivation; affective dysregulation; impulse control; social inadequacy; and psychotic symptoms. In studies conducted on patients with Parkinson's disease, a cutoff of 7.5 points or higher was adopted to distinguish subjects with MBI from those without relevant behavioral characteristics (Yoon et al., 2021).

In some studies, the MBI domains were derived indirectly from NPI/NPI-Q scores by using the algorithm proposed by Sheikh et al. (2018). Symptoms related to sleep and eating are not included in the definition of MBI but are included in the 12 traditional domains assessed by the NPI/NPI-Q.

Finally, some studies (Aarsland et al., 2009a, b; Mueller et al., 2018) have included symptoms beyond the scope of the standard domains, such as phobias, suicidal ideation, anticipatory anxiety, and compulsive behaviors. Although not all scales systematically assess these aspects, they have been considered relevant for delineating a more accurate neuropsychiatric clinical profile.

### Cognitive domains

All of the selected studies included at least one measure of cognitive functioning. Specifically, nine studies assessed global cognitive functioning, six assessed memory, five assessed language, six assessed attention, eight assessed executive functions, seven assessed visuospatial or visuomotor skills, and three assessed processing speed or visuomotor components. No additional cognitive domain was considered besides those indicated (Tables 1 and 2).

**Table 2:** Summary of the relationship between NPS and cognitive decline

Cognitive domain	Performance in PD-NPS vs. HC/PD-NPS-	Percentage of studies finding poor performance in PD NPS vs. NPS-
***Global cognitive functioning***	↓ Lee et al., 2023 ↓ Aarsland et al., 2009a, b ↓ Meng et al., 2023 ↓ Oh et al., 2021 ↓ Yoon et al., 2019 ↓ Lang et al., 2020 = Dlay et al., 2020 = Ojagbemi, 2013	6 out of 8 75%
***Memory***	↓ Lee et al., 2023 ↓ Meng et al., 2023 ↓ Yoon et al., 2019 ↓ Lang et al., 2020 = Weintraub et al., 2015 = Yoon et al., 2021	4 out of 6 67%
***Language***	↓ Lee et al., 2023 ↓ Yoon et al., 2019 ↓ Lang et al., 2020 = Kulisevsky et al., 2008 = Yoon et al., 2021	3 out of 5 60%
***Attention***	↓ Lee et al., 2023 ↓ Meng et al., 2023 ↓ Yoon et al., 2019 ↓ Lang et al., 2020 = Weintraub et al., 2015 = Kulisevsky et al., 2008	4 out of 6 %
***Executive functions***	↓ Lee et al., 2023 ↓ Meng et al., 2023 ↓ Yoon et al., 2019 ↓ Yoon et al., 2020 ↓ Lang et al., 2020 ↓ Kulisevsky et al., 2008 = Weintraub et al., 2015 = Ojagbemi, 2013	6 out of 8 67%
***Visuospatial skills***	↓ Lee et al., 2023 ↓ Meng et al., 2023 ↓ Yoon et al., 2019 ↓ Lang et al., 2020 = Weintraub et al., 2015 = Aarsland et al., 2009a, b = Kulisevsky et al., 2008	4 out of 7 57%
***Processing speed***	↓ Meng et al., 2023 ↓ Lang et al., 2020 = Weintraub et al., 2015	2 out of 3 66%

NPS = neuropsychiatric symptoms; HC = health condition ↓ = lower; ↑ = higher; = equal

### Impact of NPS on cognitive functioning

#### NPS and global cognitive functioning (*n* = 8)

Eight studies (Aarsland et al., 2009a, b; Lee et al., 2023; Meng et al., 2023; Oh et al., 2021; Yoon et al., 2019; Lang et al., 2020; Dlay et al., 2020; Ojagbemi, 2013) assessed global cognitive functioning by using instruments, such as the Mini-Mental State Examination (MMSE), the Montreal Cognitive Assessment (MoCA), and the Global Deterioration Scale. Six studies (Aarsland et al., 2009a, b; Lee et al., 2023; Meng et al., 2023; Oh et al., 2021; Yoon et al., 2019; Lang et al., 2020) found significant impairment in overall functioning in PD patients with NPS compared with those without, indicating significantly lower scores on cognitive tests. In contrast, two studies (Dlay et al., 2020; Ojagbemi, 2013) found no significant differences in overall cognitive scores between patients with and without NPS. Specifically, Dlay et al. (2020) observed no longitudinal changes in MoCA scores, whereas Ojagbemi (2013) found no associations between NPS severity and MMSE in de novo patients.

#### NPS and memory (*n* = 6)

Memory was investigated in six studies (Lee et al., 2023; Meng et al., 2023; Yoon et al., 2019, 2021; Lang et al., 2020; Weintraub et al., 2015) through tests that assessed verbal and visual memory, including the Hopkins Verbal Learning Test-Revised (HVLT-R), the Seoul Verbal Learning test, the Logical Memory subtest of the Wechsler Memory Scale-IV (WMS-IV), and the Rey-Osterrieth Complex Figure Test – recall (RCFT). Four studies (Lee et al., 2023; Meng et al., 2023; Yoon et al., 2019; Lang et al., 2020) revealed that PD patients with NPS performed significantly worse on memory tests than those without. Lower scores were particularly evident in verbal memory (Lee et al., 2023; Lang et al., 2020) and in both immediate and delayed memory (Yoon et al., 2019). However, two studies (Weintraub et al., 2015; Yoon et al., 2021) found no significant differences in memory scores between groups with and without NPS.

#### NPS and language (*n* = 5)

Five studies (Lee et al., 2023; Yoon et al., 2019, 2021; Lang et al., 2020; Kulisevsky et al., 2008) investigated language and used semantic, phonemic, and alternating fluency tests, as well as the Boston Naming Test. Three of these studies (Lee et al., 2023; Yoon et al., 2019; Lang et al., 2020) found that patients with NPS had impaired language abilities compared with those without. However, two studies (Yoon et al., 2021; Kulisevsky et al., 2008) found no significant differences in language scores between the groups.

#### NPS and attention (*n* = 6)

Six studies (Meng et al., 2023; Yoon et al., 2019; Lang et al., 2020; Lee et al., 2023; Weintraub et al., 2015; Kulisevsky et al., 2008) examined attention by using instruments, such as the Digit Span Forward of the WAIS-IV, Symbol Span of the Wechsler Memory Scale-IV, Trail Making Test A (TMT-A), and Symbol Digit Modalities Test (SDMT). Four of these studies (Meng et al., 2023; Yoon et al., 2019; Lang et al., 2020; Lee et al., 2023) reported that patients with NPS experienced worsening attention, especially when accompanied by affective, anxiety, and behavioral symptoms. These patients had difficulty with sustained attention tasks and visuomotor processing speed. In contrast, two studies (Weintraub et al., 2015; Kulisevsky et al., 2008) conducted on de novo patients found no significant differences between groups.

#### NPS and executive functions (*n* = 8)

Eight studies analyzed executive functions (Lee et al., 2023; Meng et al., 2023; Lang et al., 2020; Yoon et al., 2019, 2021; Kulisevsky et al., 2008; Weintraub et al., 2015; Ojagbemi, 2013) by using tests, such as the Trail Making Test B (TMT-B), Stroop test, Wisconsin Card Sorting Test (WCST), Clock Drawing Test (CDT), and alternating fluency. Six of these studies (Lee et al., 2023; Meng et al., 2023; Lang et al., 2020; Yoon et al., 2019, 2021; Kulisevsky et al., 2008) identified significant deficits in executive function among PD patients with NPS. In particular, lower scores were observed in cognitive flexibility, response inhibition, and verbal generation tasks. However, two studies (Weintraub et al., 2015; Ojagbemi, 2013) reported no significant differences between groups.

#### NPS and visuospatial skills (*n* = 7)

Seven studies (Meng et al., 2023; Yoon et al., 2019; Lang et al., 2020; Lee et al., 2023; Weintraub et al., 2015; Aarsland et al., 2009a, b; Kulisevsky et al. 2008) used tools, such as the Rey-Osterrieth Complex Figure Test (RCFT) copy, Judgment of Line Orientation (JOLO), Hooper Visual Organization Test (HVOT), and Benton Visual Retention Test (BVRT). Four of these studies (Meng et al., 2023; Yoon et al., 2019; Lang et al., 2020; Lee et al., 2023) reported that PD patients with NPS had lower scores on visuospatial tests than patients without NPS. Patients had difficulty with tasks requiring complex visual processing, figure reproduction, and spatial recognition. The other three studies (Weintraub et al., 2015; Aarsland et al., 2009a, b; Kulisevsky et al., 2008) found no significant differences between patients with and without NPS.

#### NPS and processing speed (*n* = 3)

Three studies (Meng et al., 2023; Lang et al., 2020; Weintraub et al., 2015) have explored processing speed by using the Symbol Digit Modalities test (SDMT) or equivalent instruments. Two of these studies (Meng et al., 2023; Lang et al., 2020) revealed that PD patients with NPS exhibited significantly slower processing speeds than those without. However, Weintraub et al. (2015), who only included *de novo* patients, found no differences in SDMT scores between groups with and without affective symptoms.

## Discussion

This review was designed to investigate the influence of NPS on cognitive functioning in patients with PD. In contrast to the numerous preceding studies that concentrated on specific symptoms, such as depression, apathy, or anxiety, this study adopted a more comprehensive approach by examining NPS in its totality. The fundamental premise of this assumption is that, in cases of PD, NPS frequently manifest in conjunction with one another rather than in isolation. This concomitant occurrence is hypothesized to exert a substantial influence on the cognitive progression of the disease.

Many studies have indicated an association between symptoms, such as apathy, depression, anxiety, and psychosis and lower cognitive performance. This association has been demonstrated in domains, including verbal memory, executive functions, and visuospatial abilities (Aarsland et al., 2009a, b; Kulisevsky et al., 2008; Lee et al., 2023). The concomitant presence of multiple symptoms or specific clusters thus appears to suggest a heightened degree of cognitive vulnerability.

Recent longitudinal research further supports the predictive value of NPS for cognitive decline over time. Meng et al. (2023) demonstrated that the combination of apathy, anxiety, and psychotic symptoms predicts significant reductions in cognitive test performance during follow-up. Similarly, studies employing the Mild Behavioral Impairment (MBI) construct (Lang et al., 2020; Yoon et al., 2019, 2021) have shown that even subtle new-onset behavioral changes correlate with measurable cognitive deterioration in early PD stages. This underscores the necessity to move beyond a fragmented view of neuropsychiatric manifestations and instead embrace their complex interplay with cognitive processes.

Most of the reviewed studies (nine out of eleven) reported worse cognitive outcomes in patients with PD and NPS compared with those without. However, two notable exceptions (Weintraub et al., 2015; Dlay et al., 2020) found no significant cognitive differences. The observed discrepancy may be attributed to methodological factors, because both studies primarily employed global cognitive screening tools, such as the MoCA. While these instruments are widely used for their efficiency and sensitivity to early deficits, they may fail to detect subtle or domain-specific impairments. A growing body of research has demonstrated that more comprehensive neuropsychological batteries, which assess memory, executive function, processing speed, and visuospatial abilities, are generally more effective in revealing cognitive differences linked to NPS (Koerts et al., 2009; Litvan et al., 2012).

The findings extend to patients newly diagnosed with PD (de novo), who, despite not yet undergoing dopaminergic treatment, already exhibit neuropsychiatric and cognitive changes. Several studies (Aarsland et al., 2009a, b; Pont-Sunyer et al., 2015; Lawson et al., 2014) confirm that NPS, such as apathy, anxiety, and depression, can serve as early markers of cognitive vulnerability in this population, supporting the hypothesis that these manifestations are an integral part of the neurodegenerative process rather than a mere side effect of therapy.

Therefore, these findings confirm that when domain-specific neuropsychological batteries are used, and the different subtypes of NPS are analyzed, a link between behavioral symptoms and cognitive decline is already detectable in the early stages of the disease. This evidence reinforces the hypothesis of a shared neuropathological substrate from the onset.

Conversely, studies that have identified a substantial correlation between NPS and cognitive function have predominantly employed more extensive neuropsychological assessment instruments, capable of evaluating specific cognitive domains, including memory, attention, language, and executive functions. This approach has facilitated the detection of reductions in verbal and visuospatial memory scores in patients with depressive symptoms (Meng et al., 2023), worsening of executive functions and attention in the presence of apathy or "mood" and "hyperactivity" clusters (Lee et al., 2023; Lang et al., 2020), and deficits in naming and visuoconstructive abilities associated with psychotic symptoms (Lee et al., 2023).

Notably, these patterns are not exclusive to PD. Similar associations between early neuropsychiatric changes and cognitive decline have been reported in populations at risk for Alzheimer’s disease, mild cognitive impairment, and multiple sclerosis, suggesting common underlying neuropathological mechanisms involving front-limbic and corticostriatal-thalamic circuits. Specific tools, such as the Mild Behavioral Impairment Checklist (MBI-C), have proven particularly valuable in detecting subtle behavioral changes that precede overt cognitive decline. Patients with elevated MBI scores consistently demonstrate poorer performance across multiple cognitive domains, also in cognitively normal populations and MCI (Blasutto et al., 2025), supporting the utility of MBI as a clinical marker of early vulnerability. This is critical for identifying patients at risk and guiding early interventions. Consequently, the implementation of domain-specific neuropsychological assessments is imperative for the timely identification of individuals at risk of cognitive decline, not only in PD but also in other neurodegenerative conditions.

Another salient issue that emerged from the review pertains to the tools employed to assess neuropsychiatric symptoms. A number of studies, including those by Yoon et al. (2019, 2021) and Lang et al. (2020), employed the Mild Behavioral Impairment Checklist (MBI-C), a questionnaire developed with the specific purpose of detecting even mild behavioral changes that may appear in the very early stages of neurodegenerative diseases. The utilization of the MBI-C in samples of Parkinson's patients without dementia is of particular pertinence, because it facilitates the detection of early indications of cognitive vulnerability when symptoms are still subtle and not clearly evident in conventional cognitive assessments. The findings from these studies indicate that patients with higher MBI-C scores demonstrate poorer performance in various cognitive domains compared with those without MBI and healthy subjects. This evidence is of great interest in the context of the present review, which included only PD patients without a diagnosis of dementia. Consequently, employing sensitive instruments such as the MBI-C is particularly advantageous, because it facilitates the early identification of patients who, despite not yet manifesting overt deterioration, already exhibit behavioral changes that could foreshadow subsequent cognitive decline.

From a neuroanatomical perspective, several studies have investigated the brain areas and circuits involved in PD patients with NPS. A recent review highlighted that patients with PD and MBI demonstrated cortical thinning and reduced volume in the right middle temporal cortex compared with those without MBI, with a correlation between the total MBI-C score and reduced cortical volume in this region (Angelopoulou et al., 2024; Yoon et al., 2019). Furthermore, compared with healthy subjects, the PD-MBI subgroup demonstrated additional cortical alterations in the left parahippocampal region, right precuneus, lateral frontal pole, and right lingual gyrus (Yoon et al., 2019). Moreover, PD patients with NPS have been found to show cortical atrophy in the temporal and frontal areas, as well as in the striatum, limbic areas, and occipital regions (Zhang et al., 2025), even in de novo patients (Szatmari et al., 2017). These results suggest the pivotal role of both the temporal and frontal cortical areas in a wide range of NPSs in PD.

However, not all results have been consistent. Some studies have reported more ambiguous or difficult-to-interpret data. This is the case, for example, with Ojagbemi, (2013), who observed a higher prevalence of psychotic symptoms, including hallucinations and agitation, in patients with MCI compared with those with normal cognitive function. However, despite this evidence, the authors failed to find a statistically significant correlation between total scores on the Neuropsychiatric Inventory (NPI) and those on the MMSE. This outcome, counterintuitive though it may be, can be attributed to several factors, including the limited sensitivity of the MMSE in detecting more subtle cognitive deficits or the poor specificity of the NPI total score, which aggregates very heterogeneous symptoms. Recent literature has underscored the limitations of generic tools, such as the MMSE, in fully capturing the complex interplay between behavioral disorders and specific cognitive functions (Hort et al., 2010).

Moreover, the study by Dlay et al. (2020), based on a 36-month longitudinal follow-up, also found no significant relationship between the severity of neuropsychiatric symptoms—assessed using the NPI-D—and cognitive decline in patients with Parkinson's disease. However, it is important to acknowledge that the primary objective of the study was not to examine the impact of NPS on cognition thoroughly but rather to describe the progression of these symptoms over time and their impact on the burden experienced by caregivers. In addition, the sole instrument employed for the cognitive assessment was the MoCA, which was administered annually as a global measure. These data suggest that, despite the absence of a significant association between behavioral symptoms and overall cognitive functioning in some studies, the clinical interest in the topic remains substantial. The absence of clear correlations may be attributable to methodological limitations rather than a genuine absence of a relationship. This observation prompts a reflection on the necessity of employing more sensitive tools and a standardized protocol to assess cognitive domains (Casagrande et al., 2022) and conducting more detailed analyses to investigate these aspects, also in clinical practice. Consequently, it would be reasonable to incorporate specific tools for evaluating NPS, such as the MBI-C or NPI, into clinical practice in conjunction with comprehensive neuropsychological batteries. In terms of research, it will be essential to promote more standardized longitudinal studies that use comparable methodologies and analyze the parallel evolution of cognitive and behavioral symptoms over time. This will allow for a more thorough understanding of the mechanisms underlying this link and the development of increasingly personalized and effective interventions.

## Limitations

It is essential to acknowledge the limitations of this review. The initial challenge pertains to the limited number of studies that met the inclusion criteria. Despite a meticulous selection process and rigorous methodological and thematic standards, only eleven articles satisfied all the stipulated requirements. Consequently, conducting a meta-analysis, a statistical technique that would have enabled a more precise estimation of the impact of NPS on cognitive function, was rendered infeasible. Consequently, the synthesis remained qualitative.

A secondary constraint pertains to the pronounced methodological heterogeneity among the studies. The instruments employed to evaluate both the NPS and cognitive functions are very dissimilar. It is also noteworthy that the majority of the included studies employed a cross-sectional design, which precludes the establishment of causal relationships between the presence of NPS and cognitive decline. However, only a limited number of studies have adopted a longitudinal approach, as evidenced by the work of Dlay et al. (2020). Nonetheless, the sensitivity of the tools utilized in these studies was found to be inadequate.

## Conclusions

Although studies are limited and methodologically variable, evidence consistently shows that a broad range of NPS are associated with poorer cognitive functioning in PD. These associations emerge both globally and within specific cognitive domains, suggesting that domain-specific assessment, especially in early stages, are more informative than global measures. Neuropsychiatric symptoms should therefore be included in comprehensive clinical evaluations, given their impact on psychological well-being, daily functioning, and risk of cognitive decline. Further research with standardized tools and larger samples is needed to clarify this relationship.

## Data Availability

Data or materials for the manuscript are available upon request.
